# A Comprehensive Review of Percutaneous and Surgical Left Atrial Appendage Occlusion

**DOI:** 10.3390/jcdd11080234

**Published:** 2024-07-27

**Authors:** Michał Święczkowski, Emil Julian Dąbrowski, Paweł Muszyński, Piotr Pogorzelski, Piotr Jemielita, Joanna Maria Dudzik, Tomasz Januszko, Małgorzata Duzinkiewicz, Maciej Południewski, Łukasz Kuźma, Marcin Kożuch, Paweł Kralisz, Sławomir Dobrzycki

**Affiliations:** 1 Department of Invasive Cardiology, Medical University of Bialystok, 24A Sklodowskiej-Curie St., 15-276 Bialystok, Poland; e.j.dabrowski@gmail.com (E.J.D.); p.muszynski94@gmail.com (P.M.); pogo.piotr@gmail.com (P.P.); pjemielita@gmail.com (P.J.); tjanuszko7@gmail.com (T.J.); magduzinkiewicz@gmail.com (M.D.); maciejpoludniewski@gmail.com (M.P.); kuzma.lukasz@gmail.com (Ł.K.); marcinkozuch@poczta.onet.pl (M.K.); paqral@yahoo.com (P.K.); kki@umb.edu.pl (S.D.); 2 Second Department of Nephrology and Hypertension with Dialysis Unit, Medical University of Bialystok, 24A Sklodowskiej-Curie St., 15-276 Bialystok, Poland; asiadudzik.jd@gmail.com

**Keywords:** atrial fibrillation, left atrial appendage occlusion, ischaemic stroke

## Abstract

Atrial fibrillation (AF) is the most common arrhythmia worldwide, and is associated with a significant risk of thromboembolic events. Left atrial appendage occlusion (LAAO) has emerged as a promising alternative for patients with contraindications or intolerance to anticoagulant therapy. This review summarises the current evidence, indications, and technical advancements in surgical and percutaneous LAAO. Preprocedural planning relies on various imaging techniques, each with unique advantages and limitations. The existing randomised clinical trials and meta-analyses demonstrate favourable results for both percutaneous and surgical LAAO. Postprocedural management emphasises personalised anticoagulation strategies and comprehensive imaging surveillance to ensure device stability and detect complications. Future focus should be put on antithrombotic regimens, investigating predictors of device-related complications, and simplifying procedural aspects to enhance patient outcomes. In summary, LAAO is presented as a valuable therapeutic option for preventing AF-related thromboembolic events, with ongoing research aimed at refining techniques and improving patient care.

## 1. Background

Atrial fibrillation (AF) remains the most common arrhythmia worldwide and its absolute number has more than doubled in the last 30 years [1]. It is projected that AF prevalence will increase from 700,000 to up to 1.8 million patients in the United Kingdom between 2010 and 2060 [2]. AF is associated with a 5-fold increase in the risk of ischaemic stroke—the most disabling complication [3]. According to research data, AF-related ischaemic stroke is nearly twice as likely to be fatal and more severe compared to non-AF stroke [4].

In 2008, anticoagulant treatment for atrial fibrillation was revolutionised with the introduction of direct oral anticoagulants (DOACs), which, due to their simplicity in dosing and lack of need for prothrombin time monitoring, became a more attractive option for patients than vitamin K antagonists [5]. However, a certain narrow group of patients who experience absolute contraindications of, intolerance to, or ineffectiveness of anticoagulant therapy still poses a great challenge for cardiologists and neurologists worldwide.

The left atrial appendage (LAA) is the most common part of the heart for thrombi formation due to slow blood flow, which occurs within the appendage because of its shape and trabeculations. According to studies, over 90% of cardiac thrombi forms in LAA in non-valvular AF [6]. Percutaneous or surgical LAA occlusion (LAAO) can be conducted as an alternative to anticoagulation in AF patients, either as a standalone or additional procedure to other heart surgery. Surgical LAAO is also speculated to serve as a solution for patients developing postoperative AF after surgery to reduce the risk of ischaemic stroke [7]. However, current guidelines are not precise in their indications, and the evidence regarding the effectiveness and safety of the methods is limited [8,9].

Although there is increasing evidence of better prognosis after LAAO, critical gaps related to the potential downsides of the procedure remain. Notably, AF accounts for less than 25% of ischaemic stroke and other factors, such as atherosclerosis, should be considered [3]. Additionally, incomplete sealing of the LAA or device-related thrombus (DRT) can result in residual ischaemic stroke risk. These concerns highlight the need for further rigorous research to confirm long-term efficacy and safety, and to better understand and mitigate these risks, as LAAO might be suitable only for selected patients.

In this review, we aim to present the current evidence, indications, technical advancements, and future perspectives regarding surgical and percutaneous LAAO.

## 2. Indications

According to ESC, increased bleeding risk is not an indication of percutaneous LAAO per se [8]. It is recommended to examine risk-score-based assessment (e.g., HAS-BLED score) to identify non-modifiable and address modifiable bleeding risk factors. Surveillance of patients with a higher risk of bleeding (HAS-BLED score ≥ 3) should be simply intensified, with earlier scheduled and more frequent review and follow-up. Moreover, modifiable risk factors should be treated. LAAO can be applied in patients with AF and contraindications for long-term anticoagulant treatment, such as active serious bleeding history, intracranial haemorrhage without a reversible cause, and associated comorbidities, such as severe thrombocytopenia < 50 platelets/uL or severe anaemia under investigation. This treatment should be considered especially if followed by non-modifiable risk factors: older age, male sex, Asian ethnicity, chronic kidney disease, cerebral disease, cerebral amyloid angiopathy, and small vessel disease. The AHA also mentions poor drug tolerance and compliance as predisposing factors [9]. The percutaneous LAAO guideline represents the IIb class of recommendation and level of evidence B due to a limited number of studies.

Surgical LAA is considered by ESC with IIb class of recommendation, C level of evidence for stroke prevention in patients with AF undergoing open heart surgery; in recent years, it is more often combined with surgical ablation of AF or as an isolated thoracoscopic procedure. Indications for this type of treatment constitute a high bleeding risk or, less frequently, contraindications for OAC. According to AHA surgical occlusion of the LAA may be considered in patients with AF undergoing cardiac surgery, as a component of the management of AF [9]. The same guidelines also suggest reduced postoperative thromboembolic events in older patients with AF history who underwent surgical LAAO. Although the class of recommendation is still weak, thanks to new evidence, recommendations increased from C to B-NR. On the other hand, according to most recent guidelines by the Society of Thoracic Surgeons, LAAO is recommended for all first-time non-emergent cardiac surgery procedures regardless of concomitant surgical ablation in patients with AF (class IA) [10].

## 3. Current Evidence

### 3.1. Randomised Clinical Trials (RCTs)

At this time, the amount of data regarding the efficacy of the left atrial appendage closure procedures, their safety, and the comparison of their effectiveness concerning the use of anticoagulant therapy in preventing ischaemic strokes and systemic embolism originating from randomised controlled clinical trials is limited. Table 1 and Table 2 present summaries of RCTs on surgical and percutaneous LAAO [11,12,13,14,15,16,17,18,19,20,21,22,23,24].

### 3.2. Meta-Analyses

Meta-analyses underscore the efficacy of surgical and percutaneous LAAO procedures, showing significant reductions in thromboembolic events, improved postoperative mortality rates, and comparable efficacy to DOACs. Furthermore, percutaneous LAAO demonstrates superiority over placebo and antiplatelet therapy (APT) demonstrates comparable efficacy to DOACs. However, there is a notable gap in the literature regarding direct comparisons between percutaneous and surgical LAAO procedures. Additionally, findings highlight the need for further research to compare the outcomes and safety profiles of these techniques directly, which could provide valuable insights into optimising treatment decisions and improving patient outcomes in atrial fibrillation management. Table 3 presents summaries of meta-analyses regarding surgical and percutaneous LAAO [25,26,27,28,29].

## 4. Percutaneous LAAO

In the early 2000s, the first devices enabling percutaneous LAAO were introduced. Later, the less invasive method was proven to be effective: the NCDR LAAO Registry and EWOLUTION registry reported 98.3% and 98.5% rates of successful procedures, respectively [30,31]. Moreover, studies have shown that high technical success can be achieved even by low-volume operators, which suggests that the learning curve could be considered to be relatively low [32,33].

Antibiotic prophylaxis, administered mostly intravenously, is given both before and after the procedure. The choice of antibiotic typically involves a cefazolin or vancomycin/clindamycin (in case of allergy to cephalosporins) [34]. The procedure itself is usually performed through the right femoral vein, which facilitates the appropriate puncture of the interatrial septum, through which the device will be introduced into the left atrium and then to the appendage. If there is no access through the right femoral vein, the left or, depending on the operator’s experience and skills, other superior vascular access, such as the left axillary vein, can be used [35]. To achieve therapeutic anticoagulation, unfractionated heparin is used. Transseptal puncture (TSP) should be performed in the lower, posterior part of the interatrial septum so that the access sheath will be positioned coaxially to the LAA. After performing TSP, the occluder is delivered under the control of transoesophageal echocardiography (TOE) or intracardiac echocardiography (ICE). Adequate sealing confirmed by imaging, as well as device stability (tug test), should be applied. In patients diagnosed with LAA thrombus, the LAAO procedure requires special caution and cerebral protection devices, such as the Sentinel cerebral protection system (Claret Medical, Inc., Santa Rosa, CA, USA), should be considered [36].

### 4.1. Available Devices

The first device used was PLAATO (ev3 Inc., Plymouth, MN, USA), which is composed of a nitinol metal cage with multiple outwardly bent struts and is covered with polytetrafluoroethylene membrane [37]. Currently, it is not commercially available and has been replaced by subsequent generations of occluders. The leading systems are Watchman FLX (Boston Scientific, Marlborough, MA, USA) and Amplatzer Amulet (Abbott, Green Oaks, IL, USA). Other devices that are available and approved in Europe are WaveCrest (Biosense Webster, Inc., Irvine, CA, USA) and LAmbre (Lifetech Scientific Co., Shenzhen, China).

Watchman is a system that has been in development for over 20 years, as the first implantation took place in 2002. Watchman 2.5 (Boston Scientific, USA) has undergone multiple clinical trials that have demonstrated its non-inferiority to DOACs [21,22,38]. The next generation of the device, Watchman FLX, was introduced in 2020. It has the shape of a parachute and is made of an 18-strut nitinol frame. It is covered with a membrane of polyethylene terephthalate, which prevents the formation of clots and encourages endothelization [39]. Stability is ensured by 12 fixation anchors in two rows, and safe manoeuvring is ensured by the ball-shaped end. Sizes from 20 to 35 mm are available. The selected size should be 10–30% larger than the LAA dimension. In addition to the difference in size (20–35 mm in FLX vs. 21–33 mm in 2.5) and the number of anchors (12 in FLX vs. 10 in 2.5), the most important change is the ability to partially recapture the device, which allows for greater precision during the procedure. The effectiveness and safety of Watchman FLX have been confirmed by the Pinnacle FLX study [40].

Recently, a steerable delivery sheath (SDS) was introduced, showcasing promising results in preliminary studies, especially in patients with severely enlarged LA. Utilization of SDS for Amulet occluder was characterised by significantly lower incidences of residual patent LAA (26% vs. 72%; *p* = 0.005), peridevice leaks (PDL) (16% vs. 64%; *p* = 0.004), and off-axis device position (0% vs. 27%; *p* = 0.02) in comparison to the standard group, which suggests better LAA sealing [41].

Another of the leading devices available on the market is the Amplatzer Amulet, the successor to the Amplatzer Cardiac Plug (ACP). The main feature that distinguishes it from Watchman is dual seal structure in the form of a disk connected to a lobe [42]. It is made of nitinol mesh. Stability is ensured by wires on the lobe, the number of which has been increased compared to ACP. Moreover, eight sizes from 16 to 34 mm are available.

### 4.2. Device Comparison

Continuous registries show similar effectiveness of the procedure regardless of the device used, and selecting the appropriate one remains a subject of debate and research. Currently, studies are comparing Amulet with Watchman 2.5, such as the Amulet-IDE trial, but few studies compare it with Watchman FLX [43]. The 3-year Amulet-IDE trial, in which 1878 patients were randomised 1:1, showed similar results in terms of implantation effectiveness and safety of use. There was no significant difference in the comparison of stroke, systemic embolism, bleeding, and mortality rates. In a meta-analysis including 19 studies, the incidence of DRT and PDL above 5 mm was significantly lower in the ACP/Amulet group compared to the Watchman group [44]. However, it is worth underlining that this study included a comparison of different generations of devices.

The first same-generation comparison was performed by Galea et al. conducting the Randomized Clinical Trial SWISS-APERO. A total of 221 patients were randomised, of which 111 were implanted with Amulet, 25 with Watchman 2.5, and 85 with Watchman FLX [24]. The Amulet group had a higher procedural complications rate (9.0% versus 2.7%; *p* = 0.047), but also lower PDL rates after 45 days (13.7% versus 27.5%, *p* = 0.02) [24]. However, after a one-year follow-up, no significant differences were found, and the incidence of CVD, ischaemic stroke, SE, and cerebrovascular events was comparable in both groups [45].

Summarising these studies, we can conclude that the effects of implantation of both leading occluders are comparable, and the possible selection of a specific device can be based on the size and morphology of the LAA, as Amulet should work better in the case of a thrombus filled or shallow appendages due to its construction and release mechanism. Despite their high effectiveness and relatively low rate of complications, the need to create a system that will clearly and safely allow for the immediate discontinuation of anticoagulant treatment necessitates further development of both devices. The first implantation of Watchman FLX PRO (Boston Scientific, USA) has already been carried out; it is coated with a new fluoropolymer called HEMOCOAT and intended to accelerate endothelialisation and more effectively prevent the formation of clots [46]. This is also the reason for developing other devices that are currently in the testing phase and may be commercially available in some time.

## 5. Surgical Closure

Surgical LAAO techniques were first introduced in 1948 by John Madden. At first, the technique was performed as an addition to Cox Maze procedures for atrial fibrillation [47]. The surgical options include epicardial suture, endocardial suture, ligation loops, resection and suture, surgical stapler, or closure devices [47]. The most essential post-procedure outcome is the effectiveness of closure, defined as a lack of flow or residual leak lower than 10 mm. The unsuccessful closure remains an independent risk factor for stroke and systemic thromboembolism in patients with AF [47].

All included techniques decrease the risk of stroke and systemic embolism [17,48]. However, the most preferable procedures with the highest success rate include resection and closure devices [48]. In the Left Atrial Appendage Occlusion Study (LAAOS), the success rates were 73% for surgical excision, 23% for suture exclusion, and 0% for stapler exclusion in a mean of 8.1 ± 12 months TOE follow up [48,49]. Comparable with other techniques, the surgical excision was associated with a lower risk of stroke or transient ischaemic attack (TIA). The sutures or staples closing the orifice gradually eroded through the wall of the appendage, allowing it to reopen. Importantly, LAAO did not significantly prolong cardiopulmonary bypass time or increase postoperative complications, including bleeding and AF. The learning curve was reported to be relatively short (after four cases, the success rate increased from 43% to 87%) [48].

In a pivotal LAAOS III trial, the participants were randomised to LAA closure mainly with exclusion and closure techniques or devices (AtriClip- AtriCure, Inc., Mason, OH, USA), and the procedure’s success was confirmed intraoperatively. The additional techniques were applied when the initial procedure was unsuccessful, resulting in a final 100% success rate [17]. The LAAOS III showed that the procedure is effective (decreasing the risk of stroke and TIA) and does not affect the bypass time, cross-clamp time, or the risk of reoperation for bleeding within 48 h after surgery [17]. However, the LAAOS III included patients with AF and two or more points in CHADS_2_–VA_2_Sc undergoing cardiac surgery on cardiopulmonary bypass, and as for now the results are limited only to this population. Furthermore, LAAO in this study was not an alternative to anticoagulation [9,17]. Most patients were prescribed anticoagulants at discharge (83.4%), and most continued to take them during follow-up (75.3%) [17]. Such high adherence to the anticoagulation was achieved due to excellent anonymisation for participants and their primary care and cardiovascular specialist [50]. However, LAAO should not be performed routinely in the population of patients undergoing cardiac surgery because it does not significantly influence the risk of stroke or mortality, and it could increase the risk of postoperative atrial fibrillation (POAF) in patients who underwent LAA closure (68.6% vs. 31.9% *p* < 0.001) [27,51].

Another population that would benefit from this procedure includes patients undergoing surgical ablation for AF. In those patients, LAAO reduced the risk of stroke (0.95 vs. 1.9%; OR 0.46; *p* = 0.005) and all-cause mortality (1.9 vs. 5%; OR 0.38; *p* = 0.0003) without effect on postoperative AF and reoperation for bleeding [52]. In another analysis, the LAAO as a single procedure did not affect the 30-day all-cause mortality, but when combined with surgical ablation it resulted in a statistically significant 35% survival benefit (OR: 0.65; *p* = 0.025), with preservation of the beneficial effect at long-term follow-up (6 years; OR: 0.61 *p* < 0.001). However, addition of surgical ablation was associated with higher risk of respiratory complications, a return to the intensive care unit, or longer hospitalisation [53]. As a result, the Society of Thoracic Surgeons (STS) clinical practice guidelines of 2017 suggest performing concomitant procedures of surgical LAA excision or exclusion in conjunction with surgical ablation for AF (a Class IIA recommendation; level of evidence C) [52]. Additionally, the novelisation of guidelines from 2024 further upgraded that recommendation to class IA [10]. Table 4 presents surgical LAAO techniques [54].

### Hybrid Closure

Hybrid endocardial/epicardial methods involve the use of a LARIAT+ device (SentreHEART Inc., Redwood City, CA, USA). The LARIAT+ device consists of a 15 mm compliant occlusion balloon catheter, 0.035 in. magnet-tipped guidewires and a 12-F suture delivery device [55,56].

The procedure starts with opening of the pericardial and transseptal spaces, placement of endocardial magnet-tipped guidewire with a balloon in LAA, connection of an endocardial magnet-tipped guidewire with an epicardial magnet, moving the suture over the LAA neck, inflation of the balloon for better stabilisation and localisation, initial tightening of the pre-tied suture, deflation of the balloon, removal of devices, and final tightening and cut-off of the suture [55,57].

The first experiments performed in a canine model showed complete LAA exclusion in all cases with consequent LAA atrophy and endothelialisation of the LAA orifice by Hybrid Closure [58]. The first off-label human feasibility LARIAT system study was performed by Bartuś et al. in 2011 in 13 patients undergoing mitral valve surgery, or catheter ablation for atrial fibrillation without significant periprocedural complications [59].

The initial proposed indications included a CHADS_2_ score ≥ 2 or CHADS_2_-VA_2_Sc score ≥ 3, contraindications or intolerance to standard OAC therapy, or failure of OAC therapy [60].

The early results showed no device-related complications and 3–5% access-related complications, with a high closure rate at 90 days (95–100%) [60]. The initial complications rate was decreased by the introduction of a Long Micropuncture Needle and standard periprocedural use of colchicine [61,62]. The most promising results show a 91% reduction in the risk of Large Pericardial Effusion when using the Long Micropuncture Needle for epicardial access [61].

In a study involving 153 patients from four centres, from whom 108 (70.6%) patients had undergone LARIAT LAA exclusion and 45 (29.4%) were excluded from the procedure and served as a control arm, the LARIAT in over 6 years mean follow-up was connected with lower thromboembolic and bleeding events rate and mortality [63]. Compared with the Watchman device, at 1-year follow-up, the LARIAT device had a lower rate of leaks, without superiority in reduction of cerebrovascular accident [64].

Another feasibility study showed that LAA ligation with hybrid methods as an addition to electrical isolation of the pulmonary veins (PVs), not only decreases the risk of thromboembolic events but can also increase the effectiveness of preserving sinus rhythm. It allows for mechanical and electrical isolation of the LAA and more extensive ablation of the left lateral ridge [56,65]. That finding lead to initiation of a percutaneous alternative to the Maze procedure for the treatment of persistent or long-standing persistent atrial fibrillation (aMAZE trial). However, the aMAZE trial at 1-year, 2-year and 5-year follow-up showed that LARIAT LAA exclusion as an addition electrical isolation of PVs increased the chance of maintaining sinus rhythm without affecting stroke incidence, survival, and quality of life [66,67].

## 6. Procedural Planning and Technique

**Figure 1:**
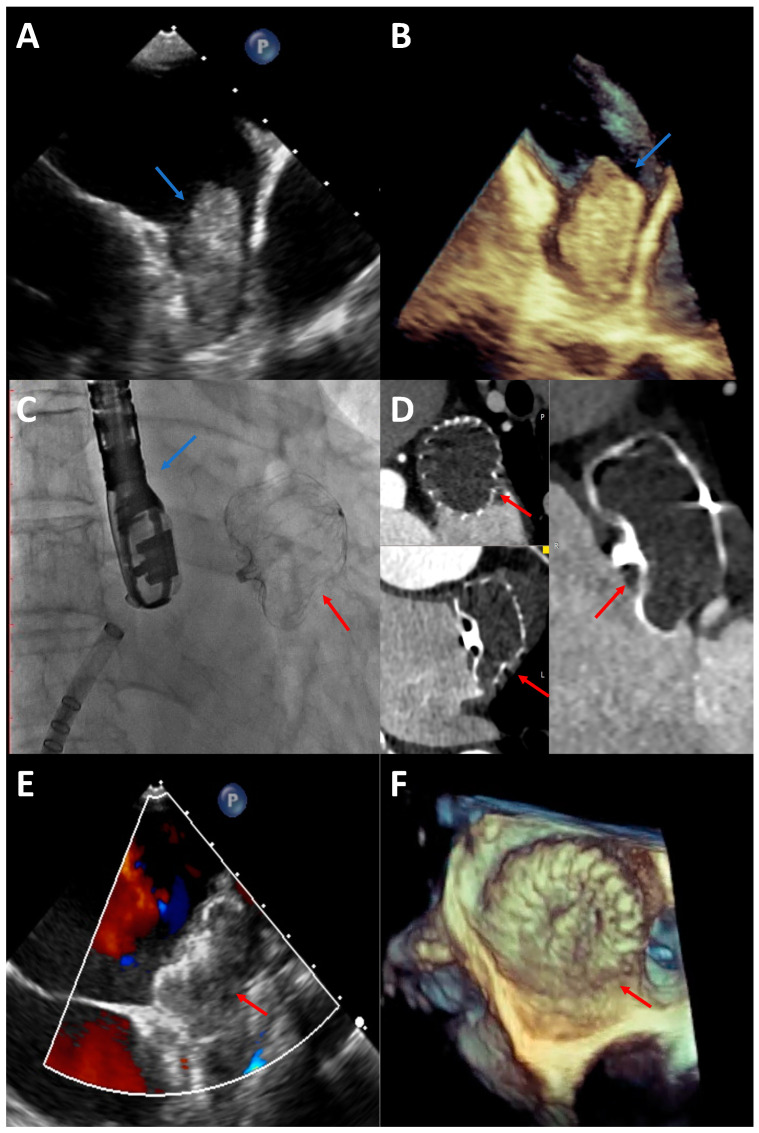
Imaging methods. (**A**) TOE—2D image. LAA with a thrombus (blue arrow). (**B**) TOE—3D image. LAA with a thrombus (blue arrow). (**C**) Intraoperative DSA. LAA occluder (red arrow) and TOE probe (blue arrow). (**D**) Follow-up CCT. LAA occluder (red arrows). (**E**) Follow-up TOE—2D image. LAA occluder (red arrow). (**F**) Follow-up TOE—3D image. LAA occluder (red arrow). Abbreviations: CCT—computer tomography; DSA—digital subtraction angiography; LAA—left atrial appendage; TOE—transoesophageal echocardiography.

Procedure planning requires high-quality pre-intervention imaging, allowing for the proper device choice, sizing, and exclusion of contraindications. TOE remains the gold standard. However, the multimodality approach includes adding cardiac computer tomography (CCT) or cardiac magnetic resonance (CMR). The current recommendation suggests TOE or CCT for preprocedure assessment and TOE or intracardiac echocardiography for intraprocedural imaging guidance [68]. Selected imaging methods are presented in Figure 1.

### 6.1. TOE

TOE, using two-dimensional (2D) and three-dimensional (3D) imaging, allows for the preprocedural exclusion of thrombi, distinguishing between various anatomy types (chicken wing, cactus, cauliflower, or windsock, and number of secondary lobes) and proper device sizing (measurement of LAA orifice diameter and depth) [47,69]. The significant additive of this technique is Doppler velocity measurement, which allows for the assessment of LAA function. The limitations include the possibility of false positive detection of thrombi, which can be caused by large pectinate muscle [69]. The sensitivity and specificity for detecting thrombus can be further increased by using echocardiographic contrast [69]. Other useful additions to performing the preprocedure TOE include volume loading with 500 mL intravenous bolus of normal saline, which not only increases measurement accuracy but also is relatively safe [70]. During the procedure, TOE guides adequate atrial septal puncture, enables proper device positioning, and allows us to identify complications when the percutaneous approach is used. However, patients’ tolerance to prolonged transoesophageal echocardiography assessment is low, and it requires periprocedural anaesthesia, which can increase the risk of the procedure, especially in fragile, elderly patients [47,69].

### 6.2. Digital Subtraction Angiography (DSA)

Digital subtraction angiography is performed with a femoral venous approach with an A 5-F marked pigtail catheter. The LAA is reached by transseptal puncture, and the sheath is placed in the ostium with RAO 20–30° with cranial angulation of 20° [71]. The LAA can be measured using computerised quantitative analysis after calibration with a 20 mm distance on the marked pigtail as a calibration reference [71]. The achieved values are comparable to those obtained using other methods, such as TOE or computer tomography [72]. However, DSA requires the exclusion of thrombus before the procedure by TOE or CT and the number of performed angiographies was found to correlate with the postprocedural MRI-detected acute brain lesions [71,73].

### 6.3. ICE

ICE may be an alternative to TOE for imaging during the procedure, decreasing the need for anaesthesia and decreasing the time of the procedure [74]. It allows confirmation of the absence of an LAA thrombus, facilitating transseptal puncture, confirming the position and stability of the device, ruling out significant peridevice leak, and monitoring for complications [74]. However, it has an increased cost compared with TOE [75,76]. Some studies show that there is a higher risk of pericardial effusion with or without tamponade [75], without an effect on the incidence of iatrogenic atrial septal defect [77]. Others show no difference in technical and procedural success rates, with no significant difference in major periprocedural or access-related complications [74,78]. Adding ICE catheters with 3D imaging further increases the accuracy and efficiency of LAAO device implantation [79]. However, importantly, ICE cannot be used for LAA measurement and device sizing. It requires an additional form of preprocedure imagining, usually cardiac CT [80]. ICE can be an alternative to TOE in patients with absolute and relative contraindications to TOE (oesophagus diseases, coagulopathy, thrombocytopenia, and a history of gastrointestinal bleeding) as many of these comorbidities are considered to be indications for LAAO [81]

ICE requires vascular access by an 8F or 10F sheath, but it can be placed in proximity to the femoral vein access for the LAAO devices, and both can be closed with a single figure-eight suture [82]. The most efficient and quality imaging can be achieved by allocating the probe in the left atrium and left superior pulmonary vein [82].

### 6.4. CCT

The CCT is a useful tool allowing for proper sizing of devices and disclosure of LAAO contraindications with effectiveness comparable to the TOE and minimal invasiveness [83]. It enables the precise visualisation of LAA anatomy and surrounding structures such as the circumflex artery or left superior pulmonary vein [47]. Regarding LAA thrombus detection, the CT has high sensitivity and negative predictive value, with a specificity of around 79%. However, it can be further increased by delayed acquisition up to 100% at a 6 min delayed phase to the angiographic phase [83]. Most LAA assessment protocols include pre-CT fluid preload with 500 mL administered orally or intravenously and should include the delayed phase (minimally 1 min) [84].

CCT shows the superiority of TOE regarding the assessment of the epicardial surroundings and thoracic cavity anatomy. That is especially beneficial for hybrid LAAO techniques [84]. Furthermore, it can be a great alternative to TOE at preprocedure assessment when a patient has contraindications to TOE (oesophageal diseases), but it cannot replace TOE in the periprocedure phase (ICE can be implemented as mentioned above).

### 6.5. CMR

The preprocedural CMR allows LAA anatomy and thrombus assessment. In comparison to DSA, CMR tends to overestimate the maximum diameter of the landing zone. However, the other diameters have good to excellent correlation with diameters from DSA and TOE [85]. Additional studies are necessary to create a CMR-standardised device chart. Available charts are based on the TOE measurements and cannot be simply transitioned into CMR measurements [85].

In a single-centre retrospective study comparing the use of TOE to CMR in preprocedural planning, there was no significant difference in thrombus exclusion, lobe count, LAA morphology, and accuracy of the predicted device size [86]. There was no difference in LAA ostial diameter, but the LAA depth was significantly larger with CMR [86].

The most promising aspect of CMR is associated with the identification of thrombi. CMR, especially delayed-enhancement CMR, is characterised by excellent sensitivity (100%), specificity (99%), and diagnostic accuracy when compared with TOE [87]. Additionally, CMR allows for the detection of high-intensity mass, which can be missed by the TOE [88]. Furthermore, it has fewer complications and contraindications when compared with TOE [89].

The limitations for CMR include the requirement of a low heart rate, preferably the presence of sinus rhythm, and an efficient respiration navigator to achieve high-quality imaging [85,90].

### 6.6. Three-Dimensional Models

Three-dimensional models of the LA and LAA can be created from CT/CMR-acquired data before LAAO procedures and 3D printed to achieve life-size physical models [84]. The 3D models allow for lesser use of contrast media, lesser device mismatch or loss, reduction of postprocedural complication [84]. Additionally, 3D-printed models can be used for teaching purposes, as they allow for LAAO to be practiced in various LAA anatomies when combined with tubes mimicking venous access [91]. Table 5 presents the preprocedural key points, while Table 6 demonstrates the advantages and drawbacks of imaging techniques.

## 7. Post LAAO Management

The 2020 ESC guidelines do not provide extensive information on the management of patients after LAAO [8]. The only recommendations are for post-implantation anticoagulant therapy and are based on device research protocols. Regardless of the device used and the risk of bleeding, patients should take 75–325 mg of acetylsalicylic acid indefinitely. However, after implantation of the Watchman device, patients are divided into groups with low and high risk of bleeding. The group with low bleeding risk should be treated with warfarin for 45 days with a target INR of 2–3 (DOACs are also possible alternatives), and then, after confirmation of proper LAA sealing, should be started on 75 mg/day of clopidogrel for up to 6 months after the procedure. On the other hand, in the Watchman group with a high risk of bleeding or in patients with an ACP/Amulet device implanted, regardless of bleeding risk, oral anticoagulants are not recommended, while clopidogrel should be immediately started at a dose of 75 mg/day for 1–6 months after ensuring LAA sealing. If the bleeding risk is very high, the duration of clopidogrel therapy may be shortened. The main goal should be to prevent side effects or, when they occur, to quickly identify and treat them. Anticoagulant therapy and imaging seem crucial. However, this part is still underexplored.

### 7.1. Anticoagulation

The majority of patients undergoing LAAO do not tolerate anticoagulation or have serious contraindications, which is the reason for attempts to de-escalate the therapy. The key is to find a balance between preventing ischaemic events and the significant increase in bleeding risk. Although the ESC recommended treatment is based on protocols from the PROTECT AF and PREVAIL trials, more strategies are used in clinical practice, and the optimal therapeutic algorithm has not been determined yet [8]. The largest comparison studies to date come from the NCDR LAAO Registry [92].

In the first study, published in 2022, among 31,994 patients who underwent successful implantation between 2016 and 2018, only 12.2% were treated with the full FDA-approved discharge and follow-up protocols from pivotal trials [93]. The most common discharge medication strategies were warfarin and aspirin (36.92%), DOAC and aspirin (20.8%), warfarin only (13.5%), DOAC only (12.3%), and dual antiplatelet therapy (DAPT, 5%). In the 45-day follow-up, the unadjusted rate of any adverse event was highest among the patients treated with warfarin and aspirin (5.7%) and lowest in the warfarin (4.0%) and DOAC (3.8%) groups. After adjustment, after 45 days and after 6 months, the risk of major or any other adverse event was significantly lower for warfarin and DOAC alone in comparison to warfarin and aspirin. There were no significant differences in the risk of any stroke or TIA, PDL above 5 mm, or DRT [93].

The second large study based on the NCDR database was published recently [92]. It included patients who were implanted with the Watchman FLX device between August 2020 and September 2021. From this group, 32,565 patients were selected who received DAPT (aspirin and clopidogrel), DOAC and aspirin, or warfarin and aspirin at discharge. To adjust for differences in baseline characteristics, for the DAPT vs. DOAC and aspirin and separately for the DAPT vs. warfarin and aspirin comparisons, 1:1 propensity score matching was performed. In the comparison of DAPT vs. DOAC and warfarin, there was no statistically significant difference in the rates of composite endpoint that involved death, stroke, major bleeding, and systemic embolism. Overall, 3.4% of patients in the DAPT group and 4.1% of patients in the DOAC and aspirin group experienced one of those events at 45 days (*p* = 0.13). The most common complication was major bleeding, which occurred significantly less frequently in the DAPT group (2.5% vs. 3.3%, *p* = 0.04) [92].

Another recently published study is the network meta-analysis of 41 studies, comparing a total of 12,451 patients [94]. Seven strategies were analysed: DOAC, VKA, single antiplatelet therapy (SAPT), DAPT (aspirin and clopidogrel), DOAC and SAPT, VKA and SAPT, and no antithrombotic therapy [94]. In the results, DOAC only was associated with a lower rate of thromboembolic and major bleeding events. This therapy also had lower mortality compared with VKA treatment. There were no differences between treatments in terms of the incidence of DRT. In comparison with SAPT, DAPT was associated with a lower incidence of thromboembolic events, without a significant difference in bleeding risk [94].

Another network meta-analysis of 10 observational studies compared short-term antithrombotic strategies. It showed that there is no significant difference among patients receiving DAPT, DOACs, and VKA in terms of stroke, DRT, and major bleeding events [95].

It is important to emphasise that the analyses included mostly observational studies, the results of which could have been influenced by underreporting, differences in patient groups, drug doses, or changes in therapy tailored to the patient. To limit the influence of these factors, the shortest possible follow-up period was selected for each study. The abovementioned analyses do not determine which strategy is optimal and the conclusion is that therapy should be tailored to the patient based on the clinical features influencing bleeding and ischaemic risk. Similar conclusions come from the prospective registry, which found that DRT was observed at various times after LAAO. It was related to patient and procedural characteristics but not to post-implantation DAPT duration [96]. Moreover, a study comparing short DAPT (<1 month), long DAPT (1–12 months), and SAPT showed no significant differences between groups in the incidence of ischaemic events [97]. However, the short DAPT strategy was associated with better outcomes, mainly due to less bleeding [97]. Similar results were obtained in another study, in which antithrombotic treatment was discontinued in one of seven selected patients within 6 months after LAAO [98]. It did not increase the risk of death or thromboembolic events after a median follow-up of 2 years. These data support the safety of shorter periods of antithrombotic therapy after LAAO in high-bleeding-risk patients [98]. The researchers went a step further by not including any anticoagulant treatment in 22/152 (14.5%) patients. No significant differences were found between patients without antithrombotic therapy and others regarding all-cause mortality or ischaemic stroke, which showed that in highly selected groups at very high bleeding risk, discontinuation of any antithrombotic therapy after LAAO might appear safe [99].

In summary, more research is definitely needed to determine the best strategy or factors that could help determine optimal therapy for each patient.

### 7.2. Imaging

To ensure that the device is properly positioned and that there is no pericardial effusion, transthoracic echocardiography should be performed before discharge. The protocol currently used for imaging tests comes from the PROTECT-AF and PREVAIL trials. In these studies, TOE was performed at 45 days, 6 months, and 12 months follow-ups to assess the stability of the device, optimal ostial position, and the possible presence and degree of residual peridevice flow [21]. Other possible complications such as embolisation, uncovered lobes, thrombus, or infection should also be assessed [47]. A less invasive method available in follow-up is CCT. It shows greater sensitivity in detecting PDL [100]. Currently, there is no evidence to determine which method is better.

## 8. Future Directions

Current European and US guidelines provide class IIb recommendations for LAAO in patients with AF and contraindications to long-term OAC therapy. Recently gathered evidence should prompt changes, especially in light of the pivotal LAAOS III trial, which proved that surgical LAAO significantly reduces the risk of stroke and systemic embolism [17]. Moreover, novel indications for LAAO should be considered in the upcoming guidelines [101]. Currently they fail to provide clear recommendations for the patients with DOAC failure, as switching between DOACs and intensification of anticoagulation remain ineffective [101]. Therefore, this group of patients appear to be good candidates for LAAO, as recent reports suggest that even those with thrombus in the LAA, previously an absolute contraindication, may now be eligible for the procedure [102]. By using cerebral embolic protection devices such as Sentinel and TriGUARD, as demonstrated by Preda et al., LAAO can be performed safely with a high success rate even in patients with thrombus or sludge in the LAA [103].

Large observational studies suggest that percutaneous LAAO as compared to DOAC may result in lower mortality rates at the price of a higher risk of major bleeding. This indicates that efforts should be put into the optimisation of a post-procedure antithrombotic regimen, as currently mostly used are treatment protocols from trials, stratification of patients into high and very-high bleeding risk and conducting further studies investigating the safety of the procedure [104]. Currently ongoing CATALYST trial (NCT04226547), comparing Amplatzer Amulet with DOAC will be adequately powered to analyse superiority for non-procedural bleeding in the LAAO arm. Occurring after 3–4% of LAAO procedures, DRT is a relatively common complication that is significantly associated with an elevated risk of ischaemic events. However, studies investigating its predictors and association with antithrombotic treatment provided conflicting results [105]. PDL is another common complication that has been associated with worse outcomes. The cut-off value for the clinically significant leak has been a matter of debate and has not yet been elucidated. A recent publication by Dukkipati et al. showed that even PDL ≤ 5 mm is related to an increased risk of thromboembolism, suggesting that future studies should investigate the outcomes and potential stroke risk reduction of PDL closures [106]. Thromboembolic event rate reduction has been reported in patients in whom LAAO was combined with catheter ablation procedures. In a multicentre registries analysis, combined procedures resulted in a 93% relative reduction in stroke and 70% in major bleeding as compared to expected rates per risk scores [107]. In the future, we should expect the simplification of procedural aspects of LAAO. Currently few studies are reporting the safety and feasibility of intracardiac echo instead of TEE-guided LAAO, contrast-less procedures, and same-day discharge procedures [106,108,109]. Moreover, studies on the new generation of DOACs are ongoing and the preliminary results are promising. Factor XI inhibitors, such as asundexian, were associated with a lower risk of bleeding and may prove to be an effective alternative for patients with intolerance to currently used DOACs [110]. Finally, future studies should also focus on clinical groups in whom LAAC may be contraindicated or cause potential harm, as suggested by Aglan et. al, who found that in patients with hypertrophic cardiomyopathy, the procedure was associated with a 90% increase in risk of stroke and an 80% increase in the risk of systemic embolism as compared to a control group treated with OAC [111].

## 9. Conclusions

LAAO seems to be a feasible alternative to anticoagulation in patients with atrial fibrillation. Percutaneous and surgical techniques offer preventive measures against thromboembolic events in patients with different profiles. Future efforts should emphasise refining closure device technologies, intraoperative and postoperative imaging, as well as antithrombotic regimens. Moreover, upcoming guidelines should focus on optimising the qualifying criteria for LAAO procedures.

## Figures and Tables

**Table 1:** RCTs about surgical LAAO.

Trial Name	Trial Type	Enrolment Period	Surgical Device	Control Arm	n	Follow-Up	CHA_2_DS_2_-VASc	HAS-BLED	Primary Endpoint	Closure, %	Results	Conclusions
LAAOS [11]	Single-centre	2001–2002	Epicardial sutures, stapler	No occlusion	77	13 ± 7 months	N/D	N/D	Safety and efficacy	66	No significant differences in cardiopulmonary bypass duration, perioperative heart failure, AF, or bleeding.	Safe at the time of CABG
Nagpal et al. [12]	Single-centre	2007	Sutures	No occlusion	43	n/d	N/D	N/D	Postoperative cerebrovascular events	82	No significant differences in CE, MI, and respiratory failure	Safe at the time of MVR
LAAOS II [13]	Multi-centre	2009–2010	Amputation or stapler	No occlusion	51	1 year	N/D	N/D	Safety and efficacy	100	At 1 year, 4 patients (15.4%) in the LAAO arm and 5 patients (20.0%) in the non-LAAO arm experienced death, MI, CE, or major bleeding (RR = 0.71; 95% CI 0.19–2.66; *p* = 0.61)	Safe at the time of cardiac surgery; positive for stroke prevention
Lee et al. [14]	Single-centre	2016	Internal ligation, Stapled Excision	Surgical Excision	28	0.4 ± 0.1 years	N/D	N/D	Safety and efficacy	43	1 patient in the internal ligation group (14%) had a stump compared with 2 (25%) in the StEx group and 3 (50%) in the SxEx group (*p* = 0.35). The overall failure rate was 57%: 63% in the internal ligation group, 60% in the StEx group, and 50% in the SxEx group (*p* = 0.85).	There was no difference in safety and efficacy among the 3 groups.
LAACS [15]	Single-centre	2010–2015	Occlusion	No occlusion	187	3.7 years	2.9 in both groups	N/D	Postoperative cerebral Ischaemic events	N/D	14 (16%) primary events in non-LAAO group vs. 5 (5%) in the LAAO group (HR = 0.3; 95% CI 0.1–0.8, *p* = 0.02). In per-protocol analysis, 14 (18%) primary events occurred in the control group vs. 4 (6%), in the LAAO group (HR = 0.3; 95% CI 0.1–1.0, *p* = 0.05).	Lower risk of ischaemic cerebral events following cardiac surgery with LAAO
Jiang et al. [16]	Single-centre	2008–2013	Sutures	No occlusion	860	N/D	N/D	N/D	Postoperative ischaemic stroke	N/D	The incidence of ischaemic stroke in LAAO group significantly lower than in non-LAAO group (0.3% vs. 4.5%, OR = 0.067, *p* < 0.001)	Lower risk of postoperative stroke during MVR with LAAO
LAAOS III [17]	Multi-centre	2012–2018	Occlusion	No occlusion	4811	3 years	4.2 in both groups	N/D	Stroke or SE	N/D	Risk of ischaemic stroke or SE lower in the LAAO group than in the non-LAAO group (HR = 0.67; 95% CI 0.53–0.85; *p* = 0.001)	Lower risk of ischaemic stroke or SE in AF patients undergoing cardiac surgery with LAAO
ATLAS Trial [18]	Multi-centre	2016–2019	Occlusion	No occlusion	562	1 year	3.4 in both groups	2.8 in LAAO, 2.9 in non-LAAO	Safety and efficiency	99	Through 1 year, 3.4% of LAAO patients and 5.6% of non-LAAO patients had thromboembolic events	Safe at the time of cardiac surgery; positive for stroke prevention

Abbreviations: AF—atrial fibrillation; CABG—coronary artery bypass graft surgery; CE—cerebrovascular events; CI; confidence intervals; HR—hazard ratio; LAAO—left atrial appendage occlusion; MI—myocardial infarction; MVR—mitral valve replacement; N/D—no data; OR—odds ratio; RCT—randomised clinical trial; RR—relative risk; SE—systemic embolism; StEx—stapled excision; SxEx—surgical excision.

**Table 2:** RCTs about percutaneous LAAO.

Trial Name	Trial Type	Enrolment Time	Used Device	Control Arm	n	Follow-Up	CHA_2_DS_2_-VASc	HAS-BLED	Primary Endpoint	Results	Conclusions
Protect AF [19]	Multi-centre	2005–2008	Watchman	Warfin	707	18 months	N/D	N/D	stroke, SE, and CV/unexplained death	3.0 primary efficacy endpoint event rate per 100 patient years (95% CrI: 1.9–4.5) in the device group and 4.9 per 100 patient years (2.8–7.1) in the control group (RR: 0.62, 95% CrI: 0.35–1.25)	LAAO is non-inferior to warfin therapy in stroke prevention.
Chun et al. [20]	Single-centre	2010–2012	Watchman	Amplatzer and Cardiac Plug	80	1 year	4.1 in Watchman group, 4.5 in control group	3.1 in both groups	Successful LAA device implantation	95% implantation success in group A (38 of 40 patients) and 100% in group B (38 of 38 patients)	High success rate of implantation of both devices.
PREVAIL [21]	Multi-centre	2010–2013	Watchman	Warfin	407	11.8 ± 5.8 months	3.8 in Watchman group, 3.9 in control group	N/D	Haemorrhagic or ischaemic stroke, SE, and cardiovascular/unexplained death	0.064 first efficacy endpoint event rate in the device group vs. 0.063 in the control group at 18 months (RR 1.07; 95% CrI: 0.57–1.89). Second coprimary efficacy endpoint (stroke or SE > 7 d after randomization) 0.025 vs. 0.020 (RR 0.005 [95% CrI: 0.019 to 0.027]	LAAO non-inferior to warfin therapy in stroke prevention
Prague-17 [22]	Multi-centre	2015–2019	Amulet/Watchman	DOAC	402	20.8 ± 10.8 months	4.7 in both groups	3.1 in LAAO group, 3 in DOAC group	Stroke, TIA, SE, cardiovascular death, major or non-major clinically relevant bleeding, or procedure-/device-related complications.	10.99% annualised rate of the primary composite outcome with LAAO vs. 13.42% with DOAC (sHR: 0.84; 95% CI: 0.53–1.31; *p* = 0.44; *p* = 0.004 for non-inferiority)	LAAO non-inferior to DOACs
Lakkireddy et al. [23]	Multi-centre	2016–2019	Amulet	Watchman	1878	12 months	4.5 in Amulet group, 4.7 in Watchman group	3.2 in Amulet group, 3.3 in Watchman group	Composite of procedure-related complications, all-cause death, or major bleeding at 12 months and composite of ischaemic stroke.	The Amulet occluder was non-inferior to the Watchman device for the primary safety end point (14.5% vs. 14.7%; RR = −0.14 [95% CI, −3.42 to 3.13]; *p* < 0.001 for non-inferiority)	The Amulet occluder was non-inferior for safety and effectiveness of stroke prevention to Watchman.
SWISS-APERO [24]	Multi-centre	2018–2021	Amulet	Watchman	221	45 days	4.2 in Amulet group, 4.4 in Watchman group	3.1 in Amulet group, 3.2 in Watchman group	The primary end point was the composite of justified crossover to a nonrandomised device during LAA closure procedure or residual LAA patency detected by CCT at 45 days	67.6% patients in Amulet group vs. 70% patients in Watchman group with primary end point occuring (RR 0.97; 95% CI 0.80–1.16; *p* = 0.713)	Amulet device non-superior to Watchman Device

Abbreviations: CCT—computer tomography; CI—confidence intervals; CrI—credible intervals; CV—cardiovascular; HR—hazard ratio; LAA—left atrial appendage; LAAO—left atrial appendage occlusion; N/D—no data; RCT—randomised clinical trial; RR—relative risk; SE—systemic embolism.

**Table 3:** Meta-analyses about percutaneous and surgical LAAO.

Authors	Year of Publication	Type and Number of Analyzed Studies	Total Number of Patients (n)	Type of LAAO	Total Number of LAAO Procedures (n)	Control Group (n)	Primary Endpoint	Secondary Endpoints	Conclusions
Mohamed et al. [25]	2021	5 RCTs	5164	Surgical	2580	2548	Stroke, TIA, or SE	All-cause mortality (1), major bleeding/or requirement of blood transfusion (2), myocardial infarction (3).	Lower risk of thromboembolic events in AF patients undergoing concomitant LAAO
Gutierrez et al. [26]	2019	4 RCTs and 18 observational studies	280,585	Surgical	36,686	243,899	Stroke/SE events incidence generally (1), in the perioperative period (2), in follow-up > 2 years (3), postoperative mortality (4).	N/A	Lower rates of embolic events and stroke in the postoperative period in patients with preoperative AF and improved survival in the mid- to long-term follow-up in patients undergoingoncomitant surgical LAAO
Kowalewski et al. [27]	2024	7 RCTs and 18 observational studies	634,774	Surgical	77,976	556,798	Early and long-term all-cause mortality and stroke incidence in non-AF and AF settings	N/A	Reduced stroke rates at early and long-term and all-cause mortality at the long-term follow-up in AF patients; no effect in non-AF patients undergoing concomitant LAAO
Al-abcha et al. [28]	2021	3 RCTs, 2 observational	4778	Percutaneous	2504	2267	Composite of stroke, SE, and cardiovascular death.	All-cause mortality (1), all major bleeding, and non-procedural major bleeding (2), all stroke (3), ischaemic stroke (4), SE (5), haemorrhagic stroke (6), CVD death (7).	Lower rate of the composite outcome of stroke, SE and cardiovascular death in the LAAO arm. Significantly lower all-cause mortality, CVD mortality, haemorrhagic stroke, major bleeding in the LAAO arm. The risk of all stroke, ischaemic stroke, and SE was similar between the two arms.
Sahay et al. [29]	2016	19 RCTs	87,831	Percutaneous	732	Warfarin 36,645, DOAC 43,314, APT 6215, placebo: 925.	All-cause mortality and stroke or SE.	Major bleeding, intracranial bleeding, and gastrointestinal bleeding.	LAAO superior to placebo and APT, and comparable to DOAC for preventing mortality, stroke or SE, with similar bleeding risk in patients with non-valvular AF.

Abbreviations: AF—atrial fibrillation; APT—antiplatelet therapy; CVD—cardiovascular disease; DOAC—direct oral anticoagulant; LAAO—left atrial appendage occlusion; RCT—randomised clinical trial; SE—systemic embolism; TIA—transient ischaemic attack.

**Table 4:** Surgical LAAO techniques.

Epicardial approach	Epicardial exclusion	Oversew
		Purse-string suture
	Exclusion through devices	Stapler
		Endoloop
		Ligasure
		TigerPaw II
		Atriclip
	Epicardial excison	Left atrial appendectomy
		Stapler
Endocardial approach	Endocardial suture ligation	
	Patch closure	
	Invagination and double-suture	

**Table 5:** Preprocedural key points.

Left atrial appendage anatomy	Appendage diameters divided into 4 basic groups: chicken wing, cactus, windsock, and cauliflower; presence of additional lobes [47].
Atrial septum	Assessment of atrial septum pathologies (e.g., septal aneurysm) [47].
Thrombus	Exclusion of thrombus [47].
Landing zone	The maximum diameter (dmax), the perimeter (p) derived diameter (dperi), the area (a) derived diameter (darea), and the minimum diameter (dmin) [85].
Angulation	Optimal LAO/RAO and cranial/caudal angulation views to direct the fluoroscopic procedure for safe puncturing of the inter-atrial septum [47].
Relation to critical structures	The left anterior descending, left circumflex arteries, and the great cardiac vein and left superior pulmonary vein [47,84].
Epicardium surroundings	Circumferential pericardial calcification and chest wall deformity [84].

**Table 6:** Advantages and drawbacks of selected imaging techniques.

	DSA	TOE	CCT	CMR	ICE
LAA measurements	++	+++	++	++	+
Thrombus detection	++	++	++	+++	++
Relation to critical structures/LAA surroundings	+	++	+++	+++	+
Invasiveness	+	+	+++	+++	+
Preprocedural assessment	+	+++	++	++	+
Periprocedural assessment	++	++	+	+	++

Abbreviations: +++–excellent; ++–good, +–poor; CCT—computer tomography; CMR—cardiac magnetic resonance; DSA—digital subtraction angiography; ICE—intracardiac echocardiography; LAA —left atrial appendage; TOE—transoesophageal echocardiography.

## Data Availability

Not applicable.
